# Differential contributions of phosphotransferases CEPT1 and CHPT1 to phosphatidylcholine homeostasis and lipid droplet biogenesis

**DOI:** 10.1016/j.jbc.2023.104578

**Published:** 2023-03-03

**Authors:** Gabriel Dorighello, Michael McPhee, Katie Halliday, Graham Dellaire, Neale D. Ridgway

**Affiliations:** 1 Departments of Pediatrics and Biochemistry & Molecular Biology, Atlantic Research Centre, Dalhousie University, Halifax, Nova Scotia, Canada; 2 Departments of Pathology and Biochemistry & Molecular Biology, Dalhousie University, Halifax, Nova Scotia, Canada

**Keywords:** CEPT1, CHPT1, phosphatidylcholine, endoplasmic reticulum, lipid droplet

## Abstract

The cytidine diphosphate-choline (Kennedy) pathway culminates with the synthesis of phosphatidylcholine (PC) and phosphatidylethanolamine (PE) by choline/ethanolamine phosphotransferase 1 (CEPT1) in the endoplasmic reticulum (ER), and PC synthesis by choline phosphotransferase 1 (CHPT1) in the Golgi apparatus. Whether the PC and PE synthesized by CEPT1 and CHPT1 in the ER and Golgi apparatus has different cellular functions has not been formally addressed. Here, we used CRISPR editing to generate *CEPT1*-and *CHPT1*-KO U2OS cells to assess the differential contribution of the enzymes to feedback regulation of nuclear CTP:phosphocholine cytidylyltransferase (CCT)α, the rate-limiting enzyme in PC synthesis, and lipid droplet (LD) biogenesis. We found that *CEPT1*-KO cells had a 50 and 80% reduction in PC and PE synthesis, respectively, while PC synthesis in *CHPT1*-KO cells was also reduced by 50%. *CEPT1* KO caused the posttranscriptional induction of CCTα protein expression as well as its dephosphorylation and constitutive localization on the inner nuclear membrane and nucleoplasmic reticulum. This activated CCTα phenotype was prevented by incubating *CEPT1*-KO cells with PC liposomes to restore end-product inhibition. Additionally, we determined that CEPT1 was in close proximity to cytoplasmic LDs and *CEPT1* KO resulted in the accumulation of small cytoplasmic LDs, as well as increased nuclear LDs enriched in CCTα. In contrast, *CHPT1* KO had no effect on CCTα regulation or LD biogenesis. Thus, CEPT1 and CHPT1 contribute equally to PC synthesis; however, only PC synthesized by CEPT1 in the ER regulates CCTα and the biogenesis of cytoplasmic and nuclear LDs.

The endoplasmic reticulum (ER) is a dynamic network of membrane tubules and sheets that are continuous with the nuclear envelope (NE) and extend to the periphery of the cell (1). The ER contains approximately 50 to 60% of cellular phospholipids, which are exported in bulk as the packaging for secretory vesicles (2) or individually by lipid transfer proteins at membrane contact sites with other organelles (3). As well, phospholipids synthesized in the ER are used for the biogenesis of lipid droplets (LDs) (4) and autophagosomes (5). The demand for phospholipids exerted by lipid packaging and export is counterbalanced by *de novo* biosynthesis in the ER of phosphatidylcholine (PC), phosphatidylethanolamine (PE), phosphatidylserine (PS) and phosphatidylinositol (PI) (6). How these biosynthetic pathways are regulated to meet the cellular demand for phospholipids, and integrated for optimal membrane composition, is poorly understood.

The *de novo* synthesis of PC, the most abundant membrane phospholipid, by the CDP-choline (Kennedy) pathway is initiated by the phosphorylation of choline. Phosphocholine is then converted to CDP-choline by CTP:phosphocholine cytidylyltransferase (CCT) α (PCYT1A) and β (PCYT1B), nuclear and cytoplasmic enzymes, respectively, that are activated by the membrane content of pathway lipid precursors and inhibited by the product PC (7). CDP-choline and diacylglycerol (DAG) are then converted to PC by choline/ethanolamine phosphotransferase 1 (CEPT1) and choline phosphotransferase 1 (CHPT1) in the ER and Golgi apparatus, respectively (8) (Fig. 1*A*). CEPT1 is a dual function enzyme that uses CDP-ethanolamine to synthesizes PE, a reaction also carried out by ethanolamine phosphotransferase 1 (EPT1/selenoprotein 1) in the Golgi apparatus (9, 10). Thus, the dual-specificity, overlapping activities, and different subcellular locations of CEPT1 and CHPT1 complicate efforts to identify the source of PC for specific regulatory and cellular functions.

**Figure 1:**
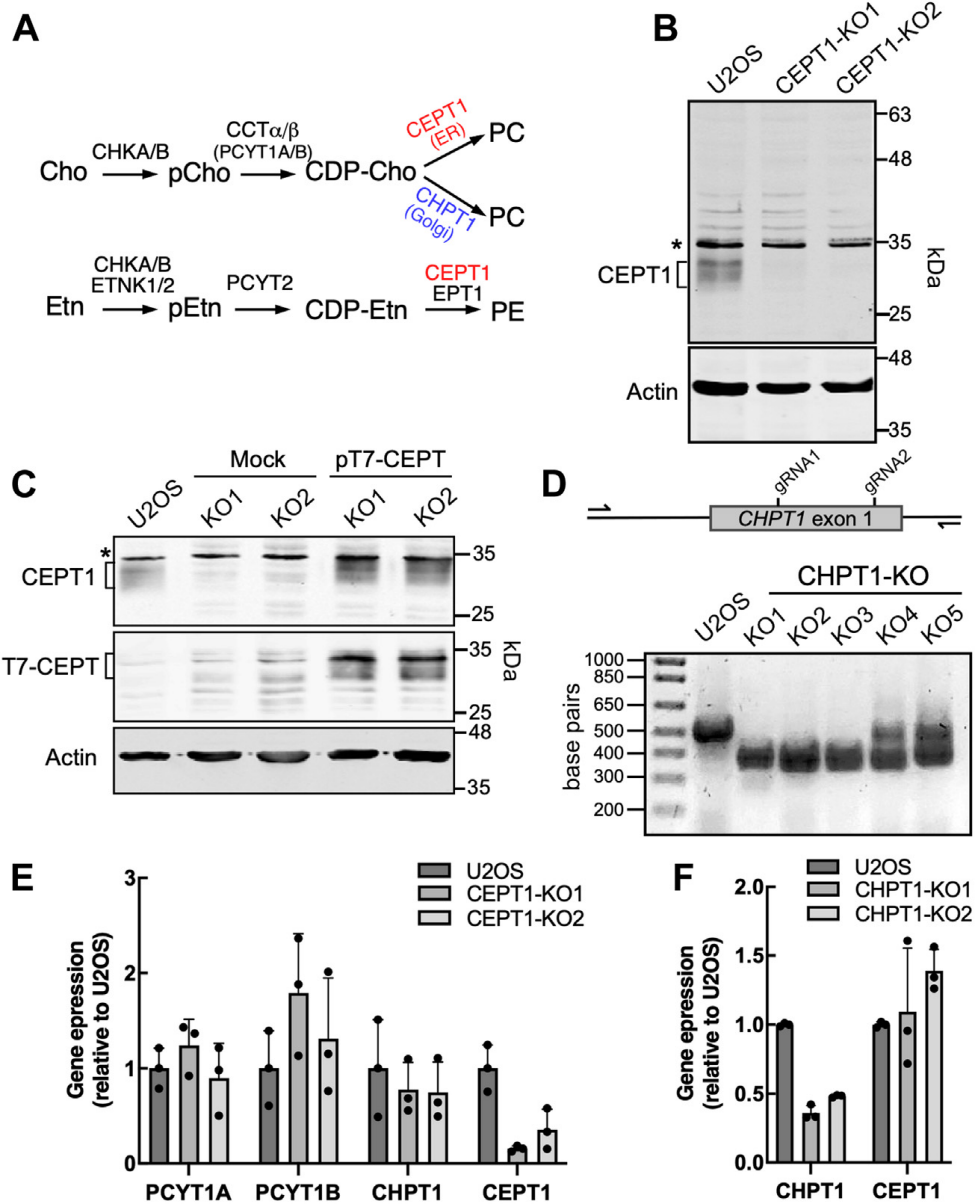
**CRISPR KO of *CEPT1* and *CHPT1* in U2OS cells.***A*, schematic of the CDP-choline (Cho) and CDP-ethanolamine (Etn) pathways. *B*, lysates of WT U2OS and two *CEPT1* KO cell lines were immunoblotted with antibodies against CEPT1 and actin. A nonspecific band is indicated by the *asterisk*. *C*, lysates of *CEPT1*-KO cells transiently transfected with empty vector (Mock) or pT7-CEPT were immunoblotted with the antibodies against CEPT1, T7, and actin. *D*, 120 bp deletion in exon 1 of *CHPT1* introduced by two gRNAs was screened by PCR amplification and agarose gel electrophoresis. *CHPT1*-KO1-3 are homozygous for the CRISPR deletion in exon 1. *E* and *F*, mRNA for CDP-choline pathway enzymes in *CEPT1*-KO (panel *E*) and *CHPT1*-KO cells (panel *F*) was quantified by qPCR analysis and expressed relative to U2OS cells. Results are the mean and SD of three biological replicates. CEPT1, choline/ethanolamine phosphotransferase 1; CHPT1, choline phosphotransferase 1.

Studies have demonstrated that the terminal enzymes in the pathway make PC and PE that is structurally unique. Compared to CHPT1, CEPT1 mRNA is ubiquitously expressed in human tissues (11, 12) and CEPT1 has greater PC biosynthetic activity based on isotope labelling after reexpression of the two enzymes in HEK293 KO cells (13). CHPT1 has a preference for synthesis of 1-alkyl species of PC (13) but how this is related to its function in the Golgi apparatus is unknown. A comparison of PE synthesis in *CEPT1*- and *EPT1*-KO HEK293 cells indicated that EPT1 is more active and the enzymes produce different PE acyl-chain species (10). At a functional level, CEPT1 and CHPT1 have been localized to PI synthase-enriched ER subdomains enriched in focal adhesion kinase interacting protein of 200 kDa and UNC-51-like kinase, components of the autophagosome initiation complex (14). CEPT1- and PI synthase-enriched domains containing Rab10 were also implicated in ER tubule extension and fusion (15). Knockdown of CCTα, CCTβ3, CEPT1, or CHPT1 expression caused inhibition of autophagy indicating that *de novo* PC and/or PE synthesis is required for membrane biogenesis during autophagosome formation (5, 16). During autophagy, PI synthase- and CEPT1-enriched regions of the ER are also associated with LDs that contain CCTβ (16). Thus triacylglycerol (TAG) packaging into LDs is coordinated with the localized synthesis of CDP-choline and PC. Whether CEPT1 and/or CHPT1 are involved in supplying PC for LD biogenesis during exposure of cell to exogenous fatty acids has not been addressed. Indeed, RNAi depletion of the CHPT1 homologue CPT in insect cells had no effect on LD biogenesis but loss of other CDP-choline pathway enzymes caused the appearance of large LDs due to a deficiency in surface monolayer PC (17).

Mouse KO studies have provided insights into the physiological roles for CEPT1 and CHPT1. Mice with a muscle-specific KO of *CEPT1* had increased PE and reduced PC mass that was linked to defective calcium uptake by the sarcoplasmic reticulum (18). The elevated PE-to-PC ratio caused increased insulin sensitivity in muscle but also susceptibility to exercise-induced muscle fatigue. A vascular endothelial cell-specific *CEPT1* KO mouse caused impaired proliferation, tubule formation, and peripheral tissue perfusion that was exacerbated in experimentally induced diabetes (19). The perfusion defect in *CEPT1*-KO mice was reversed by administration of a peroxisome proliferator-activated receptor α agonist, implicating CEPT1 as the source of the endogenous PPARα agonist 16:0 to 18:1-PC (20). Increased CHPT1 expression by super enhancer activation was linked to antiandrogen resistance in prostate cancer cells (21). However, it is uncertain whether the antiandrogen resistance conferred by increased CHPT1 expression is related to PC membrane content or a PC-derived signaling factor.

The aforementioned studies indicate that the PC produced by CEPT1 or CHPT1 in different cellular compartments could have unique structural and metabolic signaling functions. In this context, we used CRISPR edited *CEPT1*- and *CHPT1*-KO U2OS cells to examine how the spatial organization of these enzymes contributes PC that is used for (1) feedback regulation of CDP-choline pathway at the CCTα catalyzed step (22, 23, 24) and (2) assembly of cytosolic and nuclear LDs (17, 25, 26). Our results show that despite similar contributions by both enzymes to *de novo* PC synthesis, only the ER-localized CEPT1 is specifically involved in feedback regulation of nuclear CCTα and the formation of cytosolic and nuclear LDs.

## Results

### PC and PE synthesis by CEPT1- and CHPT1-KO cells

U2OS cells were chosen for this study since they express CEPT1 and CHPT1 mRNA (Fig. 1, *E* and *F*), have an active CDP-choline pathway and nuclear CCTα, and produce both cytosolic and nuclear LDs when challenged with oleate (27, 28). Immunoblotting of U2OS cell lysates revealed that CEPT1 migrated as a 30 to 34 kDa protein that was absent in two CRISPR KO cell lines (hereafter referred to as *CEPT1*-KO1 and *CEPT1*-KO2) (Fig. 1*B*). Transient expression of T7-tagged CEPT1 in *CEPT1*-KO1 and *CEPT1*-KO2 cells restored the expression of the 30 to 34 kDa protein, confirming the specificity of the CEPT1 antibody (Fig. 1*C*). KO of *CEPT1* expression resulted from a one base-pair insertion at the guide RNA (gRNA) site that caused a frame shift and premature stop codon (Fig. S1). *CHPT1* was knocked out using two gRNAs that produced a deletion in exon 1 that was identified by genomic PCR (Fig. 1*D*). Relative to U2OS cells, the expression of CEPT1 and CHPT1 mRNA in the respective KO cells was reduced by 60 to 80% (Fig. 1, *E* and *F*). *CEPT1*- and *CHPT1*-KO cells did not have a compensatory increase in CHPT1 or CEPT1 mRNA, respectively.

**Figure 2:**
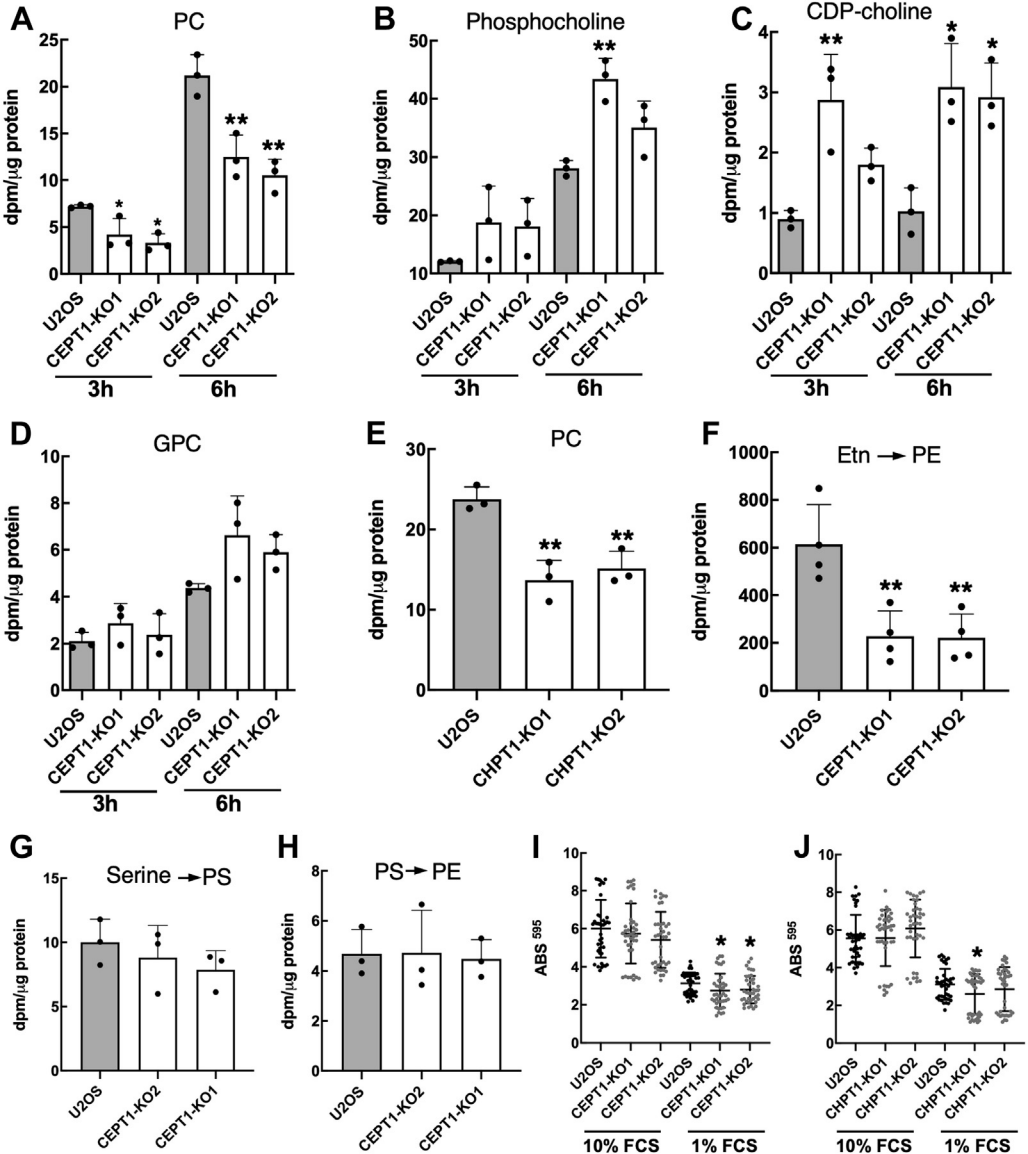
**Phospholipid synthesis in *CEPT1*- and *CHPT1*-KO cells.***A*–*D*, U2OS and *CEPT1*-KO cells were cultured in choline-free media containing 1 μCi/ml [^3^H]choline for 3 and 6 h. [^3^H]Choline incorporation into PC (panel *A*), phosphocholine (panel *B*), CDP-choline (panel *C*), and glycerophosphocholine (GPC, panel *D*) was measured relative to cellular protein. *E*, PC synthesis in *CHPT1*-KO cells was measured by [^3^H]choline labeling for 6 h. *F*, PE synthesis was measured in U2OS and *CEPT1*-KO cells by [^3^H]ethanolamine (1 μCi/ml) labeling for 3 h. *G* and *H*, PS synthesis (panel *G*) and PS decarboxylation to PE (panel *H*) in U2OS and *CEPT1*-KO cells was measure after [^3^H]serine (1 μCi/ml) labeling for 3 h in serine-free media. Results in panels *A*–*H* are the mean and SD of three biological replicates. *I* and *J*, the proliferation of *CEPT1*- and *CHPT1*-KO cells cultured in medium with 1 or 10% FCS was measured over 72 h by the crystal violet staining method (59). Results are the mean and SD of six biological replicates; significance was determined by an unpaired *t* test with matched U2OS cell controls. CEPT1, choline/ethanolamine phosphotransferase 1; CHPT1, choline phosphotransferase 1; FCS, fetal calf serum.

The effect of *CEPT1* and *CHPT1* KO on PC synthesis by the CDP-choline pathway was determined by [^3^H]choline incorporation. Compared to U2OS cells, *CEPT1*-KO1 and *CEPT1*-KO2 cells displayed a 50 to 60% reduction in [^3^H]PC synthesis after incubation with [^3^H]choline for 3 and 6 h (Fig. 2*A*). The CDP-pathway intermediates CDP-[^3^H]choline and phospho-[^3^H]choline were both increased 2-fold, consistent with a partial blockage of PC synthesis (Fig. 2, *B* and *C*). [^3^H]Choline-labeled glycerophosphocholine, a product of phospholipase degradation of PC, was not significantly affected in *CEPT1*-KO cells (Fig. 2*D*). Interestingly, two independently isolated *CHPT1*-KO cells also had a 50% reduction in [^3^H]PC synthesis (Fig. 2*E*), indicating that the two phosphotransferases contribute equally to *de novo* PC synthesis.

Synthesis of PE *via* the CDP-ethanolamine pathway is catalyzed by EPT1 as well as CEPT1 (Fig. 1*A*) (9, 10). In U2OS cells, CEPT1 is primarily responsible for *de novo* PE synthesis based on the 70% reduction in [^3^H]ethanolamine incorporation into PE observed in *CEPT1*-KO cells (Fig. 2*F*). We next assessed whether the loss of CDP-ethanolamine pathway activity in *CEPT1*-KO cells was compensated by increased PE synthesis *via* the PS synthase/PS decarboxylation pathway. However, this was not the case since the incorporation of [^3^H]serine into PS and PE was similar in U2OS and *CEPT1*-KO cells (Fig. 2, *G* and *H*). In these experiments, [^3^H]serine incorporation into PC was <5% of that for [^3^H]PE and was not affected by *CEPT1* or *CHPT1* KO (not shown), indicating that the PE methylation pathway contributes negligibly to PC synthesis in U2OS cells. Reduced PC and PE synthesis did not affect the proliferation of *CEPT1*- and *CHPT1*-KO cell cultured in normal media (10% fetal calf serum [FCS]) but there was a slight but significant growth reduction in medium containing 1% FCS (Fig. 2, *I* and *J*).

**Figure 3:**
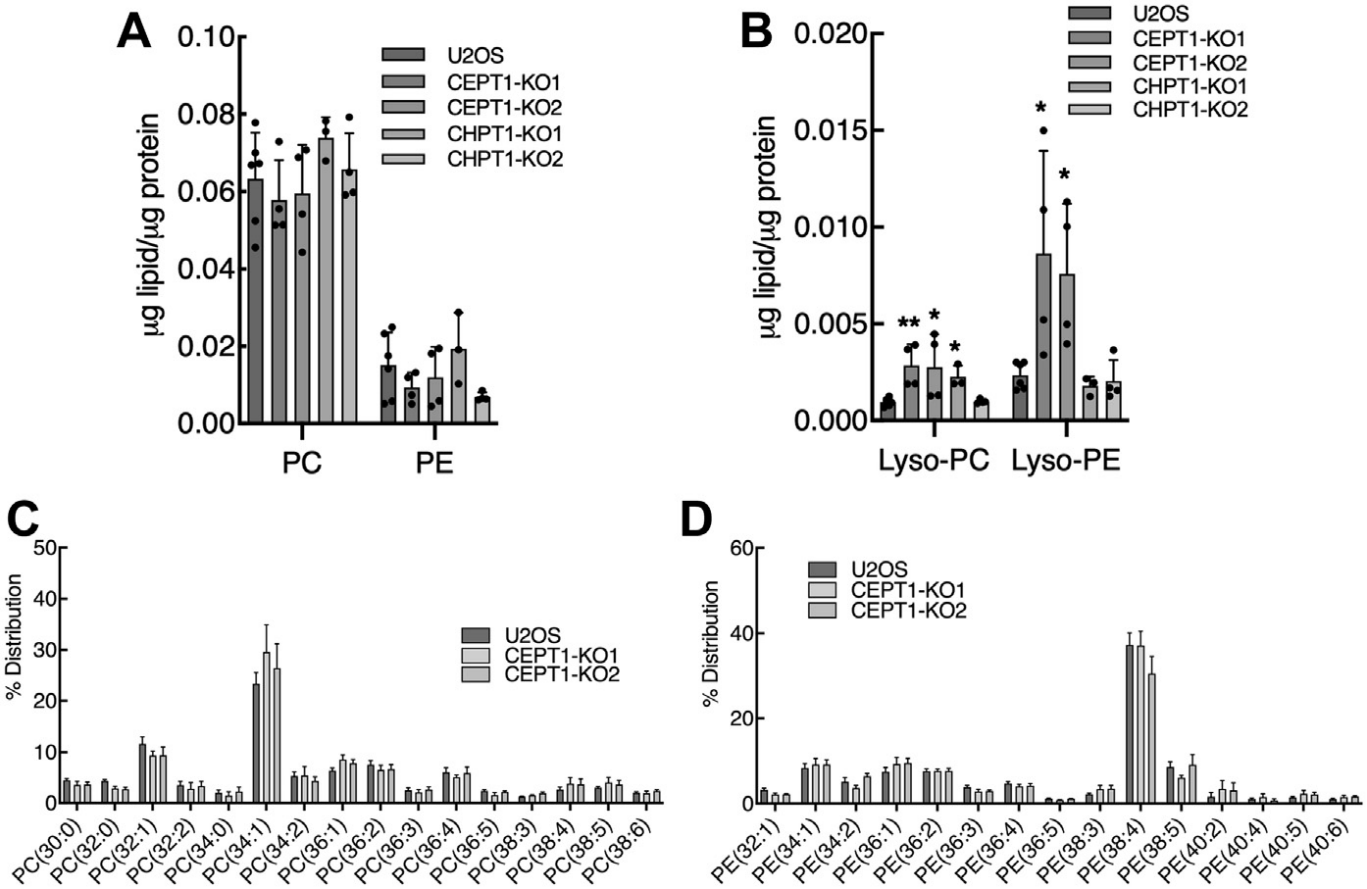
**Lipidomic analysis of U2OS, *CEPT1*-KO, and *CHPT1*-KO cells.***A* and *B*, total PC, PE, and lyso-lipids were quantified as described in the Experimental procedures. *C* and *D*, the major molecular species of diacyl-PC and diacyl-PE were quantified and expressed as a percent of total. Results are the mean and SD of 3 to 6 biological replicates; significance was determined by unpaired *t* tests with U2OS cell controls. CEPT1, choline/ethanolamine phosphotransferase 1; CHPT1, choline phosphotransferase 1; PC, phosphatidylcholine; PE, phosphatidylethanolamine.

To determine if loss of PC and PE biosynthetic activity affected cellular phospholipid mass, we conducted lipidomic analysis of *CEPT1*- and *CHPT1*-KO cells. The mass of PC and PE was unaffected in *CEPT1*- and *CHPT1*-KO cells (Fig. 3*A*); however, lyso-PE and lyso-PC mass were significantly increased in *CEPT1*-KO cells (Fig. 3*B*). The molecular species composition of PC and PE was also unaffected in *CEPT1*- and *CHPT1*-KO cells (Fig. 3, *C* and *D*), as were the molecular species of lyso-PC and lyso-PE (Fig. S2). Thus, a 50 to 70% reduction in *de novo* PC and PE biosynthetic activity does not affect the mass or molecular species composition.

### Loss of CEPT1 activity in the ER activates nuclear CCTα

**Figure 4:**
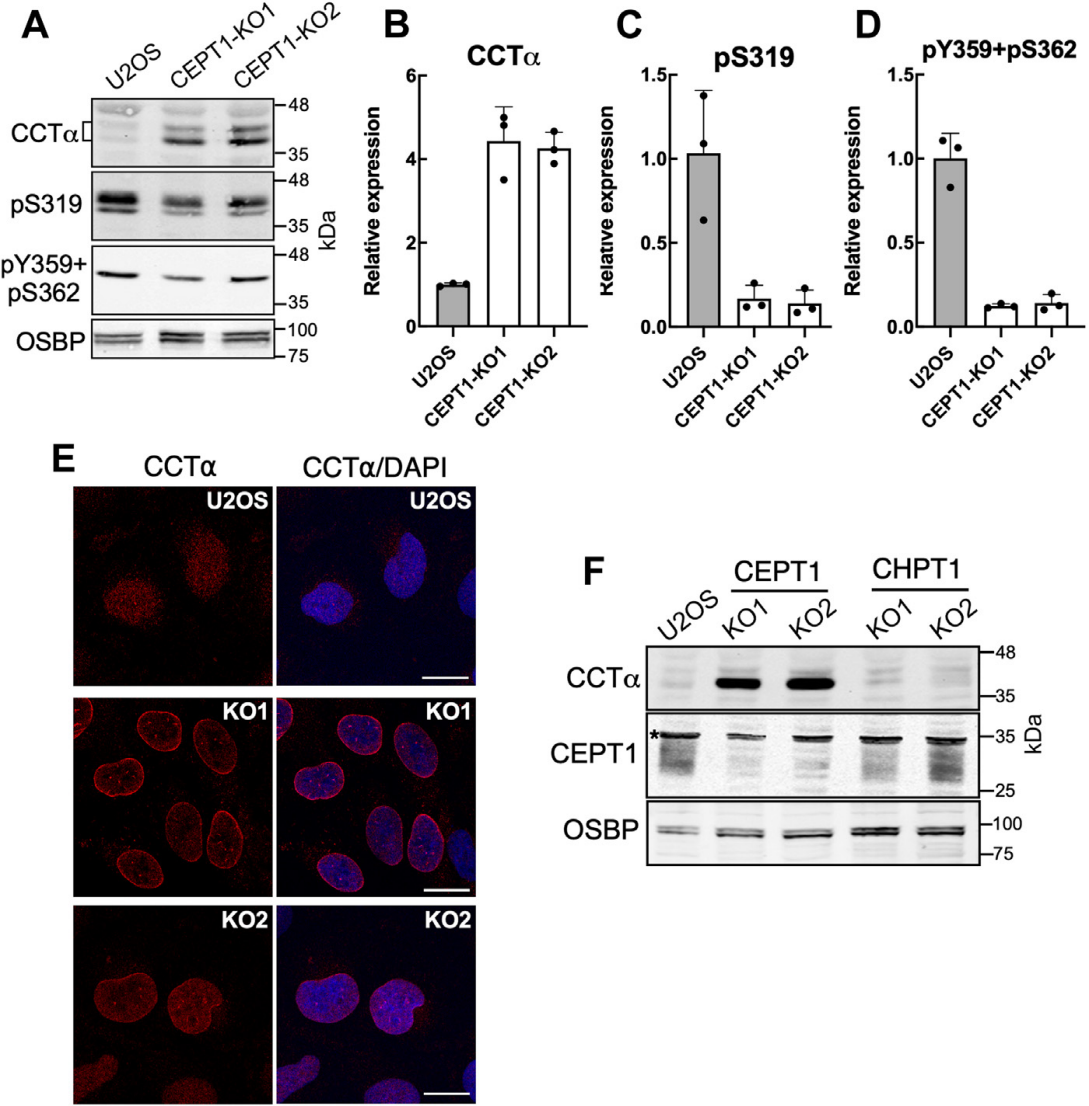
**Increased CCTα protein expression, dephosphorylation, and nuclear envelope localization in *CEPT1*-KO cells.***A*, lysates of U2OS and *CEPT1*-KO cells were immunoblotted for CCTα and the phosphosite-specific antibodies pS319 and pY359/pS362. *B*–*D*, CCTα protein expression (panel *B*) and phosphorylation of S319 (panel *C*) and Y359/S362 (panel *D*) were quantified from immunoblots. Results in panel *B* were normalized to OSBP, panels *C* and *D* were normalized to CCTα and then expressed relative to results with U2OS cells (mean and SD of three biological replicates). *E*, immunofluorescence confocal microscopy of CCTα in U2OS and *CEPT1*-KO cells (The scale bar represents 10 μm, images are representative of three independent experiments). The nucleus is stained with DAPI. *F*, lysates of *CHPT1*- and *CEPT1*-KO cells were immunoblotted for CCTα and CEPT1. CCT, CTP:phosphocholine cytidylyltransferase; CEPT1, choline/ethanolamine phosphotransferase 1; CHPT1, choline phosphotransferase 1; OSBP, oxysterol binding protein.

Activation of nucleoplasmic CCTα occurs when it translocates to membranes with (1) high negative charge density imparted by fatty acids or phosphatic acid (PA) or (2) stored curvature stress due to enrichment in nonbilayer DAG and PE and a deficiency in bilayer-forming PC (22, 23, 24, 29). Membrane translocation of CCTα is mediated by its inducible amphipathic M-domain and is accompanied by dephosphorylation of the adjacent C-terminal P-domain (28, 30, 31). Since PC is a negative regulator of CCTα due to its propensity to reduce membrane curvature stress, we tested whether loss of PC synthesis due to *CEPT1* or *CHPT1* KO would cause CCTα activation, as determined by its localization to the NE and dephosphorylation. Immunoblot analysis of cell lysates using an antibody that is insensitive to the phosphorylation status of CCTα (31) revealed that enzyme expression was increased 4-fold in *CEPT1*-KO cells compared to controls (Fig. 4, *A* and *B*). Increased CCTα protein in *CEPT1*-KO cells occurs by a posttranslational mechanism since mRNA levels were similar to U2OS cells (Fig. 1*E*). Relative to U2OS cells, phosphorylation of CCTα on S319 and Y359 + S362 was reduced by >80% in *CEPT1*-KO cells (Fig. 4, *A*, *C* and *D*), indicative of dephosphorylation of a NE-localized, activated enzyme (31). In support of this conclusion, immunofluorescence confocal imaging showed that CCTα was highly expressed and localized to the NE and nuclear puncta in *CEPT1*-KO cells (Fig. 4*E*). Despite a similar reduction in PC synthesis, CCTα expression was not increased in *CHPT1*-KO cells (Fig. 4*F*) indicating that CEPT1 alone is responsible for feedback regulation of CCTα. Collectively, the results show that CCTα is overexpressed and activated in *CEPT1*-KO cells, possibly due to reduced synthesis of PC in the ER and lack of end-product inhibition of the enzyme.

**Figure 5:**
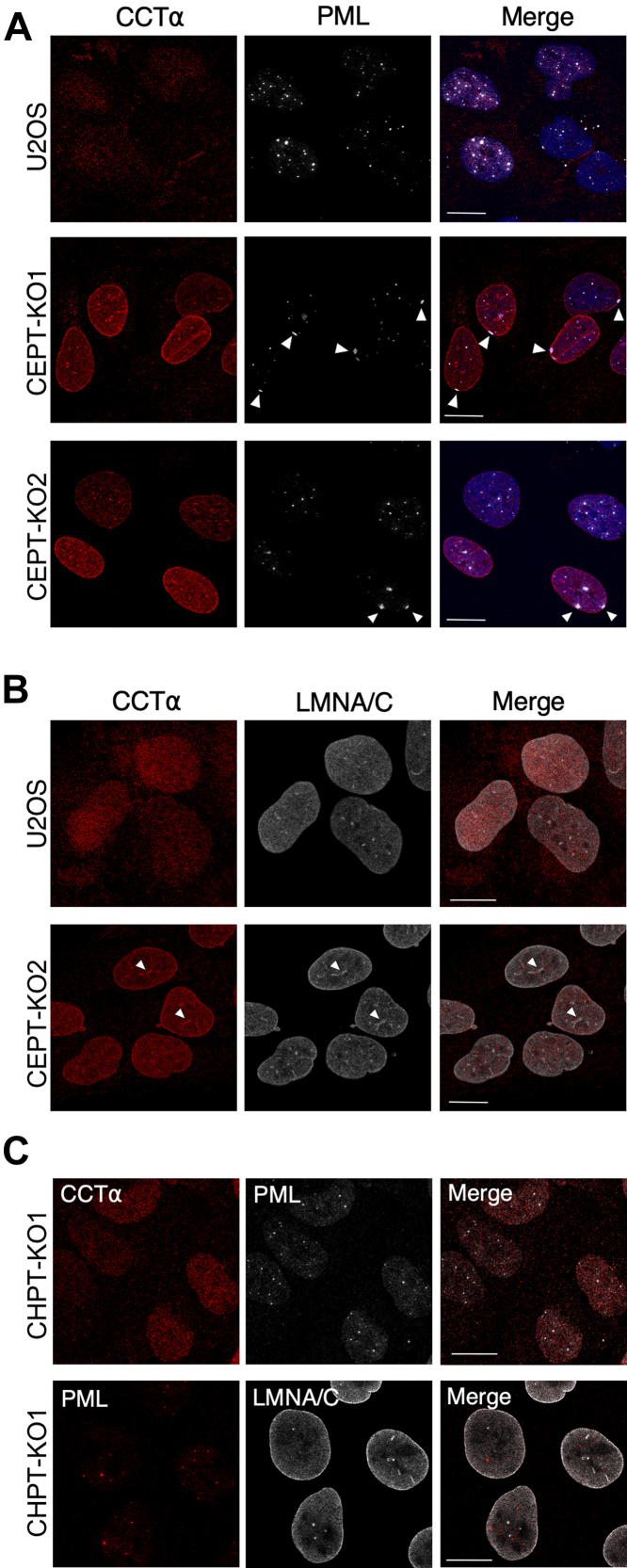
**CCTα localizes to the nucleoplasmic reticulum in *CEPT1*-KO cells.***A*, immunofluorescence confocal microscopy of U2OS and *CEPT1*-KO cells stained with antibodies against CCTα and PML. *Arrows* indicate patches of PML on the NE (bar 10 μm). *B*, immunofluorescence of U2OS and *CEPT1*-KO1 cells stained with antibodies against CCTα and LMNA/C. *Arrows* indicate localization of CCTα on NR structures (bar 10 μm). *C*, CHPT1-KO1 cells immunostained for CCTα and PML or PML and LMNA/C (The scale bar represents 10 μm). Images are representative of results from three independent experiments. CCT, CTP:phosphocholine cytidylyltransferase; CEPT1, choline/ethanolamine phosphotransferase 1; NE, nuclear envelope; PML, promyelocytic leukemia.

The CCTα-positive nuclear puncta observed in *CEPT1*-KO cells (Fig. 4*E*) could be promyelocytic leukemia (PML)-positive LDs (26). PML proteins form a nuclear scaffold called PML-nuclear bodies that mediates the posttranslational regulation of numerous transcription and chromatin remodeling factors involved in gene expression, DNA damage repair, apoptosis, and senescence (32, 33). The association of the PML and CCTα with nuclear LDs increases PC synthesis required for TAG storage in LDs (26, 28) Active CCTα also associates with and promotes the formation of NE invaginations termed the nucleoplasmic reticulum (NR) that appear as lamin-positive filaments and puncta (34, 35). Immunostaining revealed no overlap between PML-nuclear bodies and CCTα in the nucleoplasm of *CEPT1*-KO cells (Fig. 5*A*). However, *CEPT1*-KO cells had unusual patches of PML and CCTα staining on the NE (indicted by arrows) that were not evident in U2OS cells or *CHPT1*-KO1 cells (Fig. 5*C*). In U2OS and *CEPT1*-KO2 cells, lamin A/C was associated with nuclear puncta and thread-like structures (indicated by arrows) that are indicative of a type-I or type-II NR (Fig. 5*B*) (35). These NR structures also contained CCTα confirming that *CEPT1* KO leads to constitutive localization of CCTα on the NE and NR. In addition, lamin A/C-positive NR appeared to be more abundant in *CEPT1*-KO cells, an observation that is consistent with the role that activated CCTα has in forming this nuclear membrane network (34, 36). In contrast, there was no evidence of CCTα localization to the NE or NR in *CHPT1*-KO cells (Fig. 5*C*).

**Figure 6:**
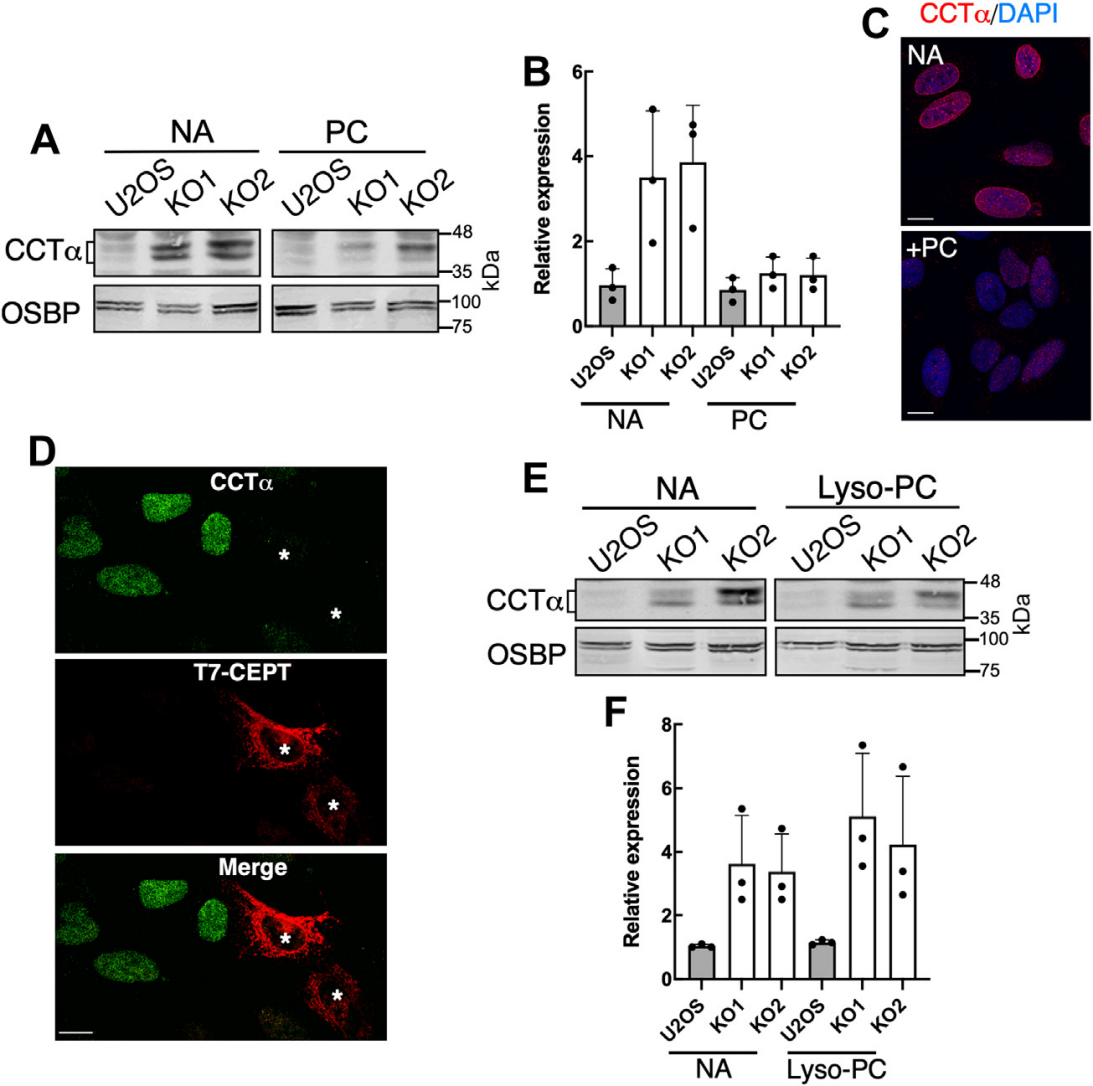
**PC supplementation of *CEPT1*-KO cells restores CCTα regulation.***A* and *B*, U2OS and *CEPT1*-KO cells were cultured in media containing no addition (NA) or PC liposomes (100 μM) for 24 h. Lysates were immunoblotted for CCTα (panel *A*), normalized to the load control (OSBP), and expressed relative to U2OS cells (panel *B*). *C*, *CEPT1*-KO cells were treated with or without PC liposomes (100 μM) for 24 and CCTα was visualized by immunofluorescence (The scale bar represents 10 μm). *D*, immunofluorescence confocal imaging of CCTα expression in CEPT1-KO cells expressing T7-CEPT1 (*asterisks*). Results are representative of two independent experiments (The scale bar represents 10 μm). *E* and *F*, *CEPT1*-KO cells were treated with lyso-PC (50 μM) or no addition for 24 h. CCTα expression in panels *B* and *F* was normalized to OSBP and expressed relative to U2OS cell controls (Results are the mean and SD of three biological replicates). CCT, CTP:phosphocholine cytidylyltransferase; CEPT1, choline/ethanolamine phosphotransferase 1; PC, phosphatidylcholine; OSBP, oxysterol binding protein.

To identify the mechanism for CCTα activation in *CEPT1*-KO cells, we tested whether supplementation of cells with PC liposomes or lyso-PC and/or lyso-PE, which are converted to PC and PE by acyltransferases, could reestablish end-product inhibition of CCTα. Culturing *CEPT1*-KO cells in media containing PC liposomes for 24 h reduced the expression CCTα to levels that were not significantly different from U2OS cells (Fig. 6, *A* and *B*). Confocal immunofluorescence of PC liposome-treated *CEPT1*-KO1 cells also confirmed that CCTα localization on the NE and NR was reduced compared to untreated cells (Fig. 6*C*). Similarly, CCTα expression was reduced in *CEPT1*-KO cells transfected with T7-CEPT1 (Fig. 6*D*). In contrast, *CEPT1*-KO cells that were incubated with lyso-PC for 24 h had the same level of CCTα expression as untreated controls (Fig. 6, *E* and *F*). The addition of lyso-PE to *CEPT1*-KO cells also failed to reduce CCTα expression relative to untreated cells and appeared to exacerbate the CCTα overexpression phenotype and promote further dephosphorylation, as indicted by the prevalence of the more rapidly migrating species on immunoblots (Fig. S3). Treatment of *CEPT1*-KO cells with a mixture of lyso-PC and lyso-PE (25 μM each) also did not reduce CCTα expression (not shown). These results indicate that PC, the product of CEPT1, restores negative feedback regulation of CCTα.

### Lack of CEPT1 causes abnormal cytoplasmic and nuclear LDs

**Figure 7:**
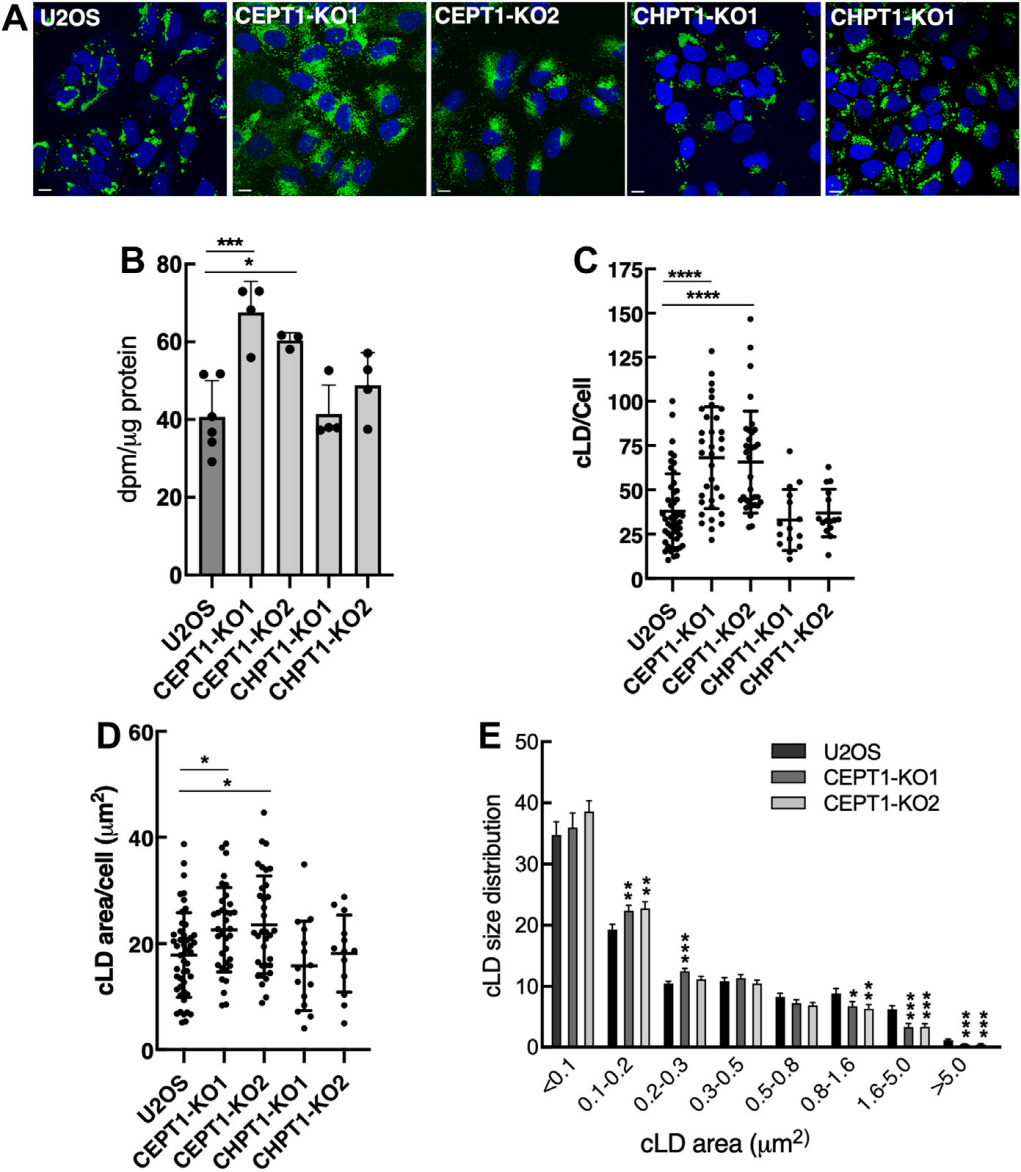
**Triglyceride synthesis and storage in *CEPT1*-KO cells.***A*, cytosolic LDs were visualized with BODIPY 493/503 in cells treated with 300 μM oleate complexed with BSA (nuclei were stained with DAPI) (The scale bar represents 10 μm). *B*, synthesis of TAG was quantified after a 4 h incubation with 100 μM [^3^H]oleate (65 nCi/ml). Results are the mean and SD of 3 to 6 biological replicates. *C*, quantification of total BODIPY-positive cLDs in U2OS and *CEPT1*-KO cells. *D*, total cLD area in U2OS and *CEPT1*-KO cells. *E*, area distribution of cLDs in U2OS and *CEPT1*-KO cells. cLDs were binned into size groupings and expressed as a percentage of total. Results in panels *B*–*D* show the mean and SD from the analysis of 15 to 40 fields of cells (representative images are shown in panel *A*) from three or four biological replicates. Significance was determined by one-way ANOVA and Tukey’s multiple comparison tests (panels *C* and *D*) or unpaired *t* tests compared to matched U2OS controls (panels *B* and *E*). BSA, bovine serum albumin; CEPT1, choline/ethanolamine phosphotransferase 1; LD, lipid droplet; TAG, triacylglycerol.

Inhibition of the CDP-choline pathway by depletion of nuclear CCT isoforms causes the formation of large cytosolic LDs due to a deficiency in surface monolayer phospholipid (37, 38, 39). However, loss of nuclear CCTα only partially blocks PC synthesis in the ER since cytoplasmic CCTβ and CEPT1 are active. We used *CEPT1*- and *CHPT1*-KO cells to investigate the consequences of complete ablation of PC synthesis in the ER and Golgi apparatus on cytosolic LD and nuclear LD biogenesis and whether CCTα would also be activated on nuclear LDs. Compared to U2OS cells, the addition of oleate to *CEPT1*-KO cells for 24 h caused the appearance of numerous small BODIPY-positive cytosolic LDs (Fig. 7*A*). This increase in oleate-induced cytosolic LD in *CEPT1*-KO cells could be attributed to a small but significant increase in TAG biosynthesis (measured by [^3^H]oleate incorporation) (Fig. 7*B*). There was no effect of *CEPT1*-KO on cholesterol ester (CE) synthesis (Fig. S4). Quantification of BODIPY-structures in Figure 7*A* showed that *CEPT1*-KO cells had a significant 50% and 30% increase in cytosolic LD number and area per cell, respectively (Fig. 7, *C* and *D*). The shift in cytosolic LD size in *CEPT1*-KO cells was confirmed by analysis of the area distribution (Fig. 7*E*), which showed a significant increase in smaller LDs at the expense of larger LDs. The results for *CEPT1*-KO cells contrast those for oleate-treated *CHPT1*-KO cells, which had a similar cytosolic LD distribution compared to U2OS cells (Fig. 7*A*) that was confirmed by quantitation of cytosolic LD number and average area (Fig. 7, *C* and *D*). Thus, similar to results for CCTα regulation, the PC produced by ER-localized CEPT1 is required for cytosolic LD biogenesis in response to oleate challenge.

**Figure 8:**
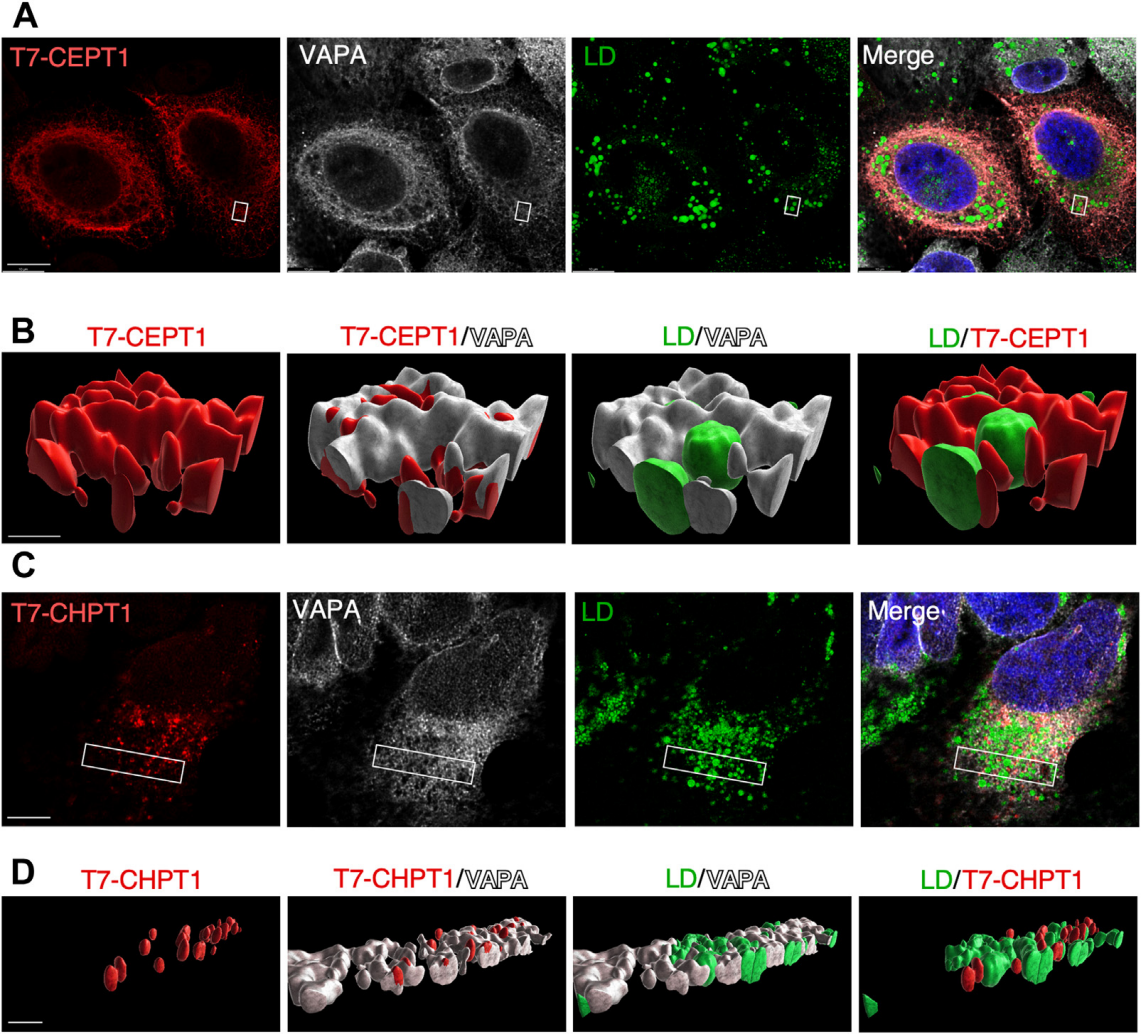
**CEPT1 localizes to the ER in proximity to cLDs.***A*, a confocal section (1 μm) from a Z-stack of CEPT1-KO1 cells expressing T7-CEPT1 and immunostained with T7 and VAPA antibodies. LDs were visualized with BODIPY 493/503 and the nuclei with DAPI (The scale bar represents 10 μm). *B*, 3D volume reconstruction of the boxed region shown in panel (The scale bar represents 1 μm). *C*, a confocal section (1 μm) from a Z-stack of CHPT1-KO1 cells expressing T7-CHPT1 immunostained as described in panel *A* (The scale bar represents 10 μm). *D*, 3D volume reconstruction of the boxed region shown in panel *C* (The scale bar represents 5 μm). Images are representative of results from two independent experiments. CEPT1, choline/ethanolamine phosphotransferase 1; CHPT1, choline phosphotransferase 1; ER, endoplasmic reticulum; LD, lipid droplet; TAG, triacylglycerol.

CEPT1 is expressed in the ER and thus could associate with and supply cytosolic LDs with PC during their biogenesis. To assess the proximity of CEPT to cytosolic LDs, *CEPT1*-KO cells expressing T7-tagged CEPT were treated with oleate to induce LD formation and 3D renderings were generated from confocal Z-stacks. On the one hand, single confocal sections revealed that T7-CEPT extensively colocalized with the ER resident protein VAPA, a tail-anchored protein that recruits lipid transfer proteins to membrane contact sites (Fig. 8*A*) (40). T7-CEPT1 was not detected on the surface of cytosolic LDs, consistent with absence of CEPT1 activity in isolated LDs (41). Three dimensional renderings of Z-stacks from a selected region (boxed in Fig. 8*A*) showed extensive colocalization of VAPA and T7-CEPT1, and frequent contacts between cytosolic LDs and sections of the ER containing VAPA and T7-CEPT1 (Fig. 8*B*). These contacts between CEPT1 and cytosolic LDs could be a conduit for delivery of PC and/or PE for the surface monolayer. On the other hand, T7-CHPT1 was localized in a punctate compartment that had minimal overlap with the ER and cytosolic LDs (Fig. 8, *C* and *D*) consistent with its lack of involvement in cytosolic LD biogenesis.

**Figure 9:**
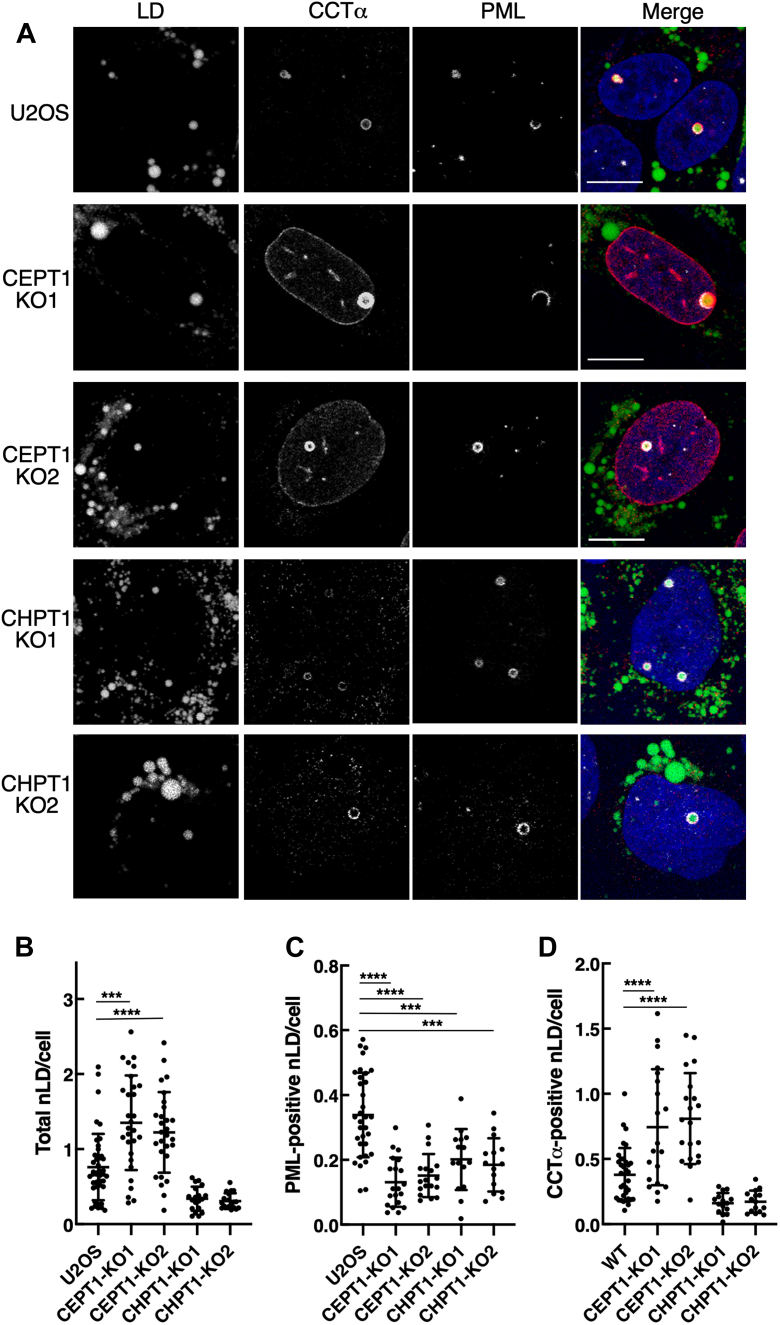
***CEPT1* KO increases nLDs enriched in CCTα.***A*, confocal immunofluorescence localization of PML and CCTα in U2OS, *CEPT1*-KO, and *CHPT1*-KO cells treated with 300 μM oleate for 24 h (The scale bar represents 10 μm). *B*, quantitation of total nuclear LDs (nLD) detected by BODIPY 493/503 staining. *C*, quantitation of PML-positive nuclear LDs (LAPS). *D*, quantitation of CCTα,positive nuclear LDs. Results in panels *B*, *C*, and *D* show the mean and SD from analysis of 15 to 50 fields of cells in three or four separate experiments (representative images are shown in panel *A*). Significance was determined by one-way ANOVA and Tukey’s multiple comparison tests. CCT, CTP:phosphocholine cytidylyltransferase; CEPT1, choline/ethanolamine phosphotransferase 1; CHPT1, choline phosphotransferase 1; LAPS, lipid-associated PML structure; LD, lipid droplet; PML, promyelocytic leukemia.

U2OS cells exposed to oleate also assemble nuclear LDs that recruit CCTα and PML on their surface (27, 28). As previously reported (28), CCTα in oleate-treated U2OS cells translocated to nuclear LDs but was absent from the NE (Fig. 9*A*). In contrast, oleate-treatment of *CEPT1*-KO1 and *CEPT1*-KO2 cells resulted in localization of CCTα to the NE and NR as well as intense staining of nuclear LDs (Fig. 9*A*). The nuclei of oleate-treated U2OS and *CEPT1*-KO cells contain a population of nuclear LDs with PML protein on their surface, termed lipid-associated PML structures (LAPS), that also harbor CCTα (Fig. 9*A*). Quantification of nuclear LDs and LAPS distribution in U2OS and *CEPT1*-KO cells showed that total BODIPY-positive nuclear LDs were increased significantly in *CEPT1*-KO cells (Fig. 9*B*). However, within the total nuclear LD pool, LAPS were decreased significantly (Fig. 9*C*) while CCTα-positive nuclear LDs were increased (Fig. 9*D*). Similar to results with cytosolic LDs, *CHPT1*-KO cells had normal levels of total nuclear LDs (Fig. 9*B*) and nuclear LD-associated CCTα (Fig. 9*D*). However, the association of PML with nuclear LDs was reduced similarly to *CHPT1*-KO cells (Fig. 9*C*). We conclude that CCTα is also absorbed onto an expanded pool of nuclear LDs in oleate-treated *CEPT1*-KO cells as a result of lack of end-product inhibition by CEPT1-derived PC.

## Discussion

The overlapping substrate specificities of the terminal enzymes in the CDP-choline and CDP-ethanolamine pathways seemingly provide redundant capacity for PC and PE synthesis (Fig. 1*A*). However, the localization of these enzymes in the ER and Golgi apparatus indicates that the PC and PE they produce is directed toward organelle-specific functions. To test this hypothesis, we used CRISPR technology to KO *CEPT1* and *CHPT1* expression in U2OS cells and then determine the individual contribution of the enzymes to *de novo* PC and PE synthesis by isotopic precursor labeling. In contrast to results with *CEPT1*-and *CHPT1*-KO HEK293 cells showing that CEPT1 was the major source of PC (13), we observed in U2OS cells that both enzymes contributed equally to PC biosynthetic capacity. *CEPT1*-KO U2OS cells also had a 70% reduction in PE synthesis from ethanolamine, which also contrasts with the 10% contribution of CEPT1 in HEK293 cells (10). These differences in PE and PC synthesis could be attributed to cell-specific variations in the expression of CEPT1 and CHPT1, as well as that of CCTα and β in the nucleus and cytoplasm, respectively, that produce substrate for CEPT1 and CHPT1. Reduced PE synthesis by the CDP-ethanolamine pathway in U2OS *CEPT1*-KO cells did not result in increased activity of the alternate PE synthase/PS decarboxylase pathway. This was not unexpected since the CDP-ethanolamine pathway is a minor biosynthetic route for PE in cultured cells (42), which instead rely on PS synthase/PS decarboxylase (43, 44). Even though PC and PE synthesis were significantly reduced in *CEPT1*- and *CHPT1*-KO cells, there was no effect on PC or PE mass or the molecular species of diacyl-PC. Similar results were observed in HEK293 cells with single and double KO of *CEPT1* and *CHPT1* (13), supporting the existence of alternate route(s) for PC synthesis, uptake of exogenous PC and/or compensatory reduction in PC degradation.

Despite similar contributions to *de novo* PC synthesis by CHPT1 and CEPT1 in U2OS cells, the arm of the CDP-choline pathway that terminates with CEPT1 in the ER is specifically involved in feedback suppression of CCTα. In untreated *CEPT1*-KO cells, CCTα was constitutively associated with the inner nuclear membrane and NR, and the P-domain was dephosphorylated, both features of CCTα activation in cells stimulated with lipid activators or depleted of PC (22, 34, 38, 45). CCTα was also strongly associated with the INM in oleate-stimulated *CEPT1*-KO cells and had enhanced association with nuclear LDs, a mechanism to increase PC synthesis for LD biogenesis and ER expansion (28, 46). The constitutive activation of CCTα in *CEPT1*-KO cells was accompanied by a 4-fold increase in enzyme protein that was not due to transcriptional induction. CCTα degradation is enhanced by mono-ubiquitination near its nuclear localization signal, which promotes retention in the cytoplasm and proteolysis in lysosomes (47, 48). Thus, enhanced nuclear membrane association of CCTα in *CEPT1*-KO cells could prevent cytoplasmic export and degradation leading to elevated enzyme expression in response to PC deficiency.

Addition of PC liposomes to *CEPT1*-KO cells was sufficient to revert membrane-association and expression of CCTα to control levels, indicating that the primary regulator is the end-product of the pathway. It is unlikely that exogenous liposomal PC was degraded by lipases to a secondary lipid regulator since most of these have stimulatory activity toward CCTα (*i.e.*, DAG, phosphatic acid, or fatty acids) and would enhance membrane association. Another approach to bypass the CDP-choline pathway is by addition of lyso-PC, which is acylated to PC by lyso-PC acyltransferases. We had successfully used lyso-PC to revert the abnormal LD phenotype due to reduced PC synthesis in Caco2 cells lacking CCTα (49). However, the addition of lyso-PC to *CEPT1*-KO cells did not affect CCTα expression and localization, possibly due to insufficient acylation to PC and/or lack of delivery to the INM where CCTα is regulated. In addition, the lack of effect of lyso-PC could be due to prexisting elevated levels of lyso-PC in *CEPT1*-KO cells (Fig. 3*B*), indicative of enhanced PC acyl-chain remodeling or scavenging from the media.

If PC is the primary lipid regulator of CCTα, why then was enzyme activation not correlated with reduced cellular PC mass? An explanation is that CCTα is regulated by the lipid composition of the INM, which constitutes a small fraction of total cellular membranes. Moreover, CCTα does not recognize the molecular features of PC *per se* but rather elastic curvature stress stored in membranes due to PC deficiency and the presence of other lipid regulators. The α-helical domain M mediates membrane association and activation of CCTα by virtue of its ability to “sense” the elastic curvature stress imparted by nonbilayer and negatively charged lipid activators, such as DAG, PE and fatty acids, and PC deficiency (7). Manipulating the content of lipid activators and PC in yeast to favor elastic curvature stress and packing defects promoted domain M membrane insertion and activation of the CCTα homologue Pct1p on the INM (22). However, yeast Pct1p is sensitive to PC produced by the methylation or CDP-choline pathways and its localization to the INM is strongly correlated with cellular PC content. In the case of *CEPT1*-KO cells, we propose that CHPT1 activity supplies sufficient PC for critical functions but the INM remains chronically PC-deficient due to the absence of CEPT1 and continued export of PC from the ER. This deficiency can be overcome by supplying a source of exogenous PC that replenishes the INM and restores CCTα regulation. It is also possible that a fraction of CEPT1 is localized in the INM, as was demonstrated in yeast (50), where it could produce PC that directly regulates CCTα activity.

An unexpected phenotype of oleate-treated *CEPT1*-KO cells was the biogenesis of smaller, more numerous cytosolic LDs that is suggestive of a defect in cytosolic LD maturation. This phenotype is markedly different from cells that have reduced PC synthesis as a result of CCTα depletion, which led to larger cytosolic LDs due to a deficiency in monolayer phospholipids. The key difference between the two models is that CCTα-deficient cells have intact CEPT1 but reduced PC synthesis in the ER due to limited CDP-choline synthesis, while *CEPT1*-KO cells have a complete absence of PC synthesis in the ER and reliance on the CHPT1 pathway (Fig. 1*A*). As mentioned above, a shift to CHPT1 as the sole source of PC in *CEPT1*-KO cells is sufficient to maintain growth but may become limiting under conditions where additional capacity is required, such as cytosolic LD biogenesis. An additional requirement for CEPT1 could relate to its close proximity to cytosolic LD in the ER, which would facilitate efficiently deliver of PC to the cytosolic LD monolayer. The loss of this association in *CEPT1*-KO cells could inhibit cytosolic LD maturation but the precise impact on the machinery involved in cytosolic LD biogenesis remains to be determined.

Similar to their cytoplasmic counterparts, total nuclear LDs were also increased in oleate-treated *CEPT1*-KO cells. The increased association of CCTα with nuclear LDs in *CEPT1*-KO cells is consistent with reduced PC content of the INM, the site at which nuclear LD form in U2OS cells (27). These nascent nuclear LDs would have a PC-deficient monolayer that, like the INM, would enhance the association of CCTα. The reduced number of LAPS in *CEPT1*-KO cells could also be due to increased expression of CCTα that could displace PML from nuclear LDs and/or a reduced PC content of the INM that affects PML interaction with the INM and nuclear LDs. It is noteworthy that PML was frequently associated with the INM in *CEPT1*-KO cells (Fig. 5*A*) indicative of structural irregularities in the membrane or lamina that led to PML recruitment.

In summary, CEPT1 and CHPT1 have equal contributions to *de novo* PC synthesis and either enzyme is capable of supporting cell proliferation. However, the PC produced by the enzymes is spatially separate, with CEPT1 in the ER being solely responsible for producing PC for feedback regulation of the CDP-choline pathway at the CCTα catalyzed step and the proper assembly of cytosolic LDs and nuclear LDs. The function of CHPT1 is unknown but its location in the Golgi apparatus intimates a possible role in secretory regulation by conversion of DAG, a known effector of vesicular transport (51, 52), to PC.

## Experimental procedures

### Cell culture

Human bone osteosarcoma epithelial (U2OS) cells (American Type Culture Collection, HTB-96) and U2OS-derived *CEPT1*- and *CHPT1*-KO cells were cultured in DMEM supplemented with 10% FCS. Cells were periodically tested for *mycoplasma* infection by PCR or nucleic acid imaging methods. KO cells were transfected with pT7-CEPT or pT7-CHPT1 (8) using a Lipofectamine 2000/DNA ratio of 3:1 (μl/μg) for 48 h. Oleate/bovine serum albumin (BSA) complexes (6:1 mol/mol) were prepared according to (53) and added to cells at 0.3 mM oleate to induce LD formation. PC liposomes were prepared by evaporating the solvent from 1-palmitoyl-2-oleoyl-PC (Avanti Polar Lipids Inc) in a glass tube and resuspending the phospholipid film in PBS. The lipid suspension (10 mM PC) was subjected to six 20 s pulses (on ice) using a probe sonicator set at 60 Hz and the liposomes used immediately for experiments. U2OS cells were incubated with 100 μM PC liposomes, which is ∼4 times the concentration of PC in the culture media. Cells were also treated with 1-oleoyl-lyso-PC or -PE (Avanti Polar Lipids Inc), which was dissolved in ethanol and added to culture media at final concentration of 50 μM. To confirm that lyso-PC was taken up and converted to PC in CEPT1-KO cells, lyso-[^3^H]PC (prepared by phospholipase A2 digestion of [^3^H]choline-labeled PC) was used as a tracer to monitor uptake over a 24 h continuous-pulse. Uptake of lyso-[^3^H]PC into cells was rapid and peaked at 12 h with 10 to 30% conversion to [^3^H]PC at individual time points (results not shown).

### CRISPR KO of CEPT1 and CHPT1

Oligonucleotides encoding a gRNA targeting exon 1 of *CEPT1* (TGAGTGGGCATCGATCAACA) or a pair of gRNAs targeting exon 1 of *CHPT1* (GGAGCACCGCTACAGCGCGG and GTAGGAGATGAGCACGAGCG) were annealed and cloned into pX459 linearized with Bbs1. U2OS cells were transiently transfected with pX459-gRNA constructs, selected in medium containing puromycin (1 μg/ml) for 2 days, and clonal cell lines were isolated by dilution subcloning and screened for gene KO. *CEPT1*-KO cells were identified by immunoblotting of whole cell lysates using an CEPT1 polyclonal antibody (N-14, Santa Cruz Biotechnology) (Fig. 1*B*) and verified by genomic PCR amplification and sequencing of the region encompassing the gRNA site in exon 1 (Fig. S1). The U2OS *CHPT1* gene was disrupted using two gRNAs to introduce a 128 nt deletion in exon 1, which was identified by genomic PCR amplification and verified by sequencing (Fig. 1*D*).

### Immunoblotting

Cells were scraped from dishes in cold PBS and collected by centrifugation at 3000*g* for 5 min. The cell pellet was lysed in sample buffer (62.5 mM Tris–HCl, pH 6.8, and 10% glycerol, 2% SDS, 0.05% bromophenol blue, and 5% β-mercaptoethanol), sonicated for 10 s, and heated at 90 °C for 3 min. Cells lysates for detection of endogenous CEPT1 and T7-CEPT were not heated prior to SDS-PAGE. Proteins were separated by SDS-PAGE and transferred to nitrocellulose membranes, which were then incubated with a blocking buffer consisting of Li-Cor Intercept diluted 1:5 (v/v) with TBS-Tween (10 mM Tris–HCl, pH 7.4, 150 mM NaCl, and 0.1% Tween 20). Nitrocellulose membranes were incubated in blocking buffer with the following primary antibodies: CCTα rabbit polyclonal (54); CEPT1 rabbit polyclonal (N-14, Santa Cruz Biotechnology); T7-TAG mouse monoclonal (Novagen Inc); CCTα phospho-S319 and CCTα phospho-S359/Y364 rabbit polyclonals (PN546 and PN548, Kinexus Bioinformatics); and pan-oxysterol binding protein antibody (55). Nitrocellulose membranes were subsequently incubated with IRDye 800CW and 680LT secondary antibodies (Li-Cor Biosciences). Fluorescence intensity of protein bands was quantified using a Li-Cor Odyssey imaging system and associated software (v3.0) and expressed relative to internal protein load controls (actin or oxysterol binding protein) or CCTα protein in the case of CCTα phospho-site quantitation.

### Radiolabeling of phospholipids and triglyceride

The biosynthesis of phospholipids was measured by pulse-labeling cells with [^3^H]choline (1 μCi/ml), [^3^H]ethanolamine (2 μCi/ml), or [^3^H]serine (2 μCi/ml) for the times indicated in figure legends. Briefly, cells cultured in choline-free media containing [^3^H]choline were harvested by scraping in 1 ml methanol/water (1:1, v/v) and extracted in chloroform/methanol (1:2, v/v) as described (56). The radiolabeled water-soluble intermediates in the CDP-choline pathway were separated by TLC in ethanol/water/ammonia (48/095/6, v/v), visualized with phosphomolybdic acid, and quantified by liquid scintillation counting. The organic phase containing [^3^H]PC was evaporated and quantified by scintillation counting. [^3^H]Ethanolamine-labeled PE was extracted in chloroform/methanol and quantified by scintillation counting. [^3^H]Serine-labeled PS and PE were extracted as described above, separated by TLC in chloroform/methanol/water (65/25/4, v/v), and quantified by scintillation counting. Isotope incorporation into soluble choline metabolites and phospholipids was normalized to cellular protein determined by the bicinchoninic acid (BCA) method (Pierce BCA assay kit, Thermo Fisher Scientific).

TAG and CE synthesis was determined by incubating cells with 100 μM [^3^H]oleate/BSA for 4 h. After rinsing cell monolayers twice with cold 150 mM NaCl, 50 mM Tris–HCl (pH 7.4) with 2 mg/ml BSA, and once with the same buffer without BSA, [^3^H]oleate-labeled lipids were extracted with hexane:isopropanol (3:2, v/v). The extracts were dried, separated by TLC (hexane:diethyl ether:acetic acid; 90:30:1, v/v), and radioactivity in TAG and CE was quantified by scintillation counting and normalized to total cellular protein.

### Immunofluorescence confocal microscopy

Cells seeded on glass coverslips for 24 to 48 h were subjected to treatments described in figure legends, fixed with 4% (w/v) paraformaldehyde and permeabilized for 10 min with 0.2% (w/v) Triton X-100 at 20 °C. Coverslips were blocked overnight at 4 °C in PBS containing 1% (w/v) BSA (PBS/BSA), followed by incubation with primary antibodies against CCTα (54), PML (monoclonal E-11; Santa Cruz), or lamin A/C (mouse monoclonal 4C11, Cell Signaling) overnight at 4 °C. After incubation with AlexaFluor594-or 647-conjugated secondary antibodies, coverslips were incubated with 4′,6-diamidino-2-phenylindole and BODIPY 493/503 for 30 min. Next, the coverslips were mounted in Mowiol on glass slides and imaged using a Leica TCS SP8 LIGHTNING Confocal microscope equipped with a Plan-Apochromat 63× (1.4 NA) oil immersion objective. The confocal images shown in figures are 0.8 to 1 μm sections and are representative of results from 3 to 6 independent experiments. LD cross sectional area and colocalization in confocal images was quantified using ImageJ software (https://imagej.nih.gov/ij/download.html) (v1.47, National Institutes of Health). Images were converted to 8 bit, the threshold was adjusted, and the “analyze particle” command was used to exclude cells on edges. LD area and number in the cytosol and nucleus was determined from images with and without masking the nucleus with the DAPI channel using the “region of interest” command. CCTα and PML-positivity of nuclear LDs was determined by overlay of BODIPY-stained images with the appropriate 594 or 647 channel.

For 3D rendering of T7-CEPT1 and T7-CHPT1 localization with LDs, U2OS cells were fixed and permeabilized as described above and immunostained with VAPA polyclonal (57) and T7-TAG monoclonal antibodies, BODIPY 493/503, and DAPI. Four-channel confocal Z-stacks were captured using a Leica SP8 LIGHTNING confocal microscope and subsequently imported into Imaris software (9.7.0) for 3D rendering. After selecting and cropping to regions of interest, each Z-stack channel was rendered as a separate 3D surface and the background fluorescence removed by the automatic threshold feature. All objects of each surface were assigned to the same class and given the same color. Image (tiff) snapshots of the rendered BODIPY surface objects in and around the rendered VAPA, T7-CEPT1, and T7-CHPT1 surface objects were captured using the Imaris animation feature.

### Quantitative PCR

Total RNA extracted from cultured cells with a RNeasy Mini Kit (QIAGEN) was the template for single-strand cDNA synthesis using SuperScript II RT(Invitrogen). qPCR assays containing SsoAdvanced Universal SYBR Green Supermix (BIO-RAD), cDNA template (100 ng) and forward and reverse oligonucleotide (500 nM) (Table S1) were amplified using a Mastercycler RealPlex thermocycler (Eppendorf). Results were normalized to *GAPDH* and *PGK1* gene expression and relative gene expression was quantified using the ΔΔCT method (58).

### Lipidomic analysis

Dishes of cells (1 × 10^7^) were rinsed twice in cold PBS, scraped in 1 ml of 0.1 N HCl:methanol (1:1, v/v), and an aliquot was removed for protein analysis by the BCA method. Lipids were extracted in 0.5 ml chloroform after the addition of a deuterated lipid standard (SPLASH Lipidomix, Avanti Polar Lipids), dried under nitrogen, and resuspended in mobile phase solvent. Untargeted lipidomic analysis was performed by LC-ESI-MS/MS on a Vanquish (Thermo Fisher Scientific) ultra high performance liquid chromatography system in-line with a QExactive (Thermo Fisher Scientific) orbitrap MS/MS operated in data-dependent acquisition mode. Separation of lipid extract (10 μl) was achieved by reverse phase chromatography on an Accucore (Thermo Fisher Scientific) C30 column (250 × 2.1 mm I.D., particle size: 2.8 μm) with a 4-component mobile phase gradient of acetonitrile, isopropanol, water, and 0.1% formic acid/400 mM ammonium formate. The chromatographic separation was carried out at 30 °C for 35 min with a flow rate of 0.2 ml/min. Each sample was injected in duplicate to acquire both positive and negative polarity mode datasets. The following ionization and MS/MS parameters were selected: the sheath gas, 40; auxiliary gas, 5; ion spray voltage, 3.2 kV; capillary temperature, 300 °C; mass range, 300 to 1700 m/z; full scan mode at a resolution of 70,000 m/z; top-1 m/z and stepped collision energy of 25, 35 (arbitrary unit); isolation window, 1 m/z; and automatic gain control target, 1e5. Data acquisition was performed using XCalibur 4.0 software (Thermo Fisher Scientific). Lipids were identified and quantified using LipidSearch software version 5.0. The individual data raw files were searched for product ion MS/MS spectra of lipid precursor ions. MS/MS fragment ions were predicted for all precursor adduct ions measured within ±5 ppm. The product ions that matched the predicted fragment ions within a ±5 ppm mass tolerance was used to calculate a match-score and those candidates providing the highest quality match were determined. Next, the search results from the individual positive or negative ion files from each sample group were aligned within a retention time window (±0.2 min) and the data were merged for each annotated lipid.

### Data quantification and statistical analysis

GraphPad Prism 9.0 (GraphPad Software Inc) was used for statistical analyses by unpaired *t* test or ANOVA (refer to figure legends). Bar and scatter plots show the mean and SD for the number of biological replicates indicated in figure legends and significance is denoted as: ∗∗∗∗*p* < 0.0001, ∗∗∗*p* < 0.001, ∗∗*p* < 0.01, ∗*p* < 0.05.

## Data availability

Data used in the current study are available from the corresponding author upon reasonable request.

## Supporting information

This article contains supporting information.

## Conflict of interest

The authors declare no conflict of interest with the contents of this article.
