# Supplementary material for: Differential contributions of phosphotransferases CEPT1 and CHPT1 to phosphatidylcholine homeostasis and lipid droplet biogenesis
Source: J Biol Chem. 2023 Mar 3;299(4):104578. doi: 10.1016/j.jbc.2023.104578 (PMC10166788; doi:10.1016/j.jbc.2023.104578)
Supplement: Supporting Figures S1–S4 and Table S1 [file mmc1.docx]

Differential contributions of phosphotransferases CEPT1 and CHPT1 to phosphatidylcholine homeostasis and lipid droplet biogenesis

Gabriel Dorighello^l^, Michael McPhee^l^, Katie Halliday^l^, Graham Dellaire^l,2^ and Neale D. Ridgway^l^*

**
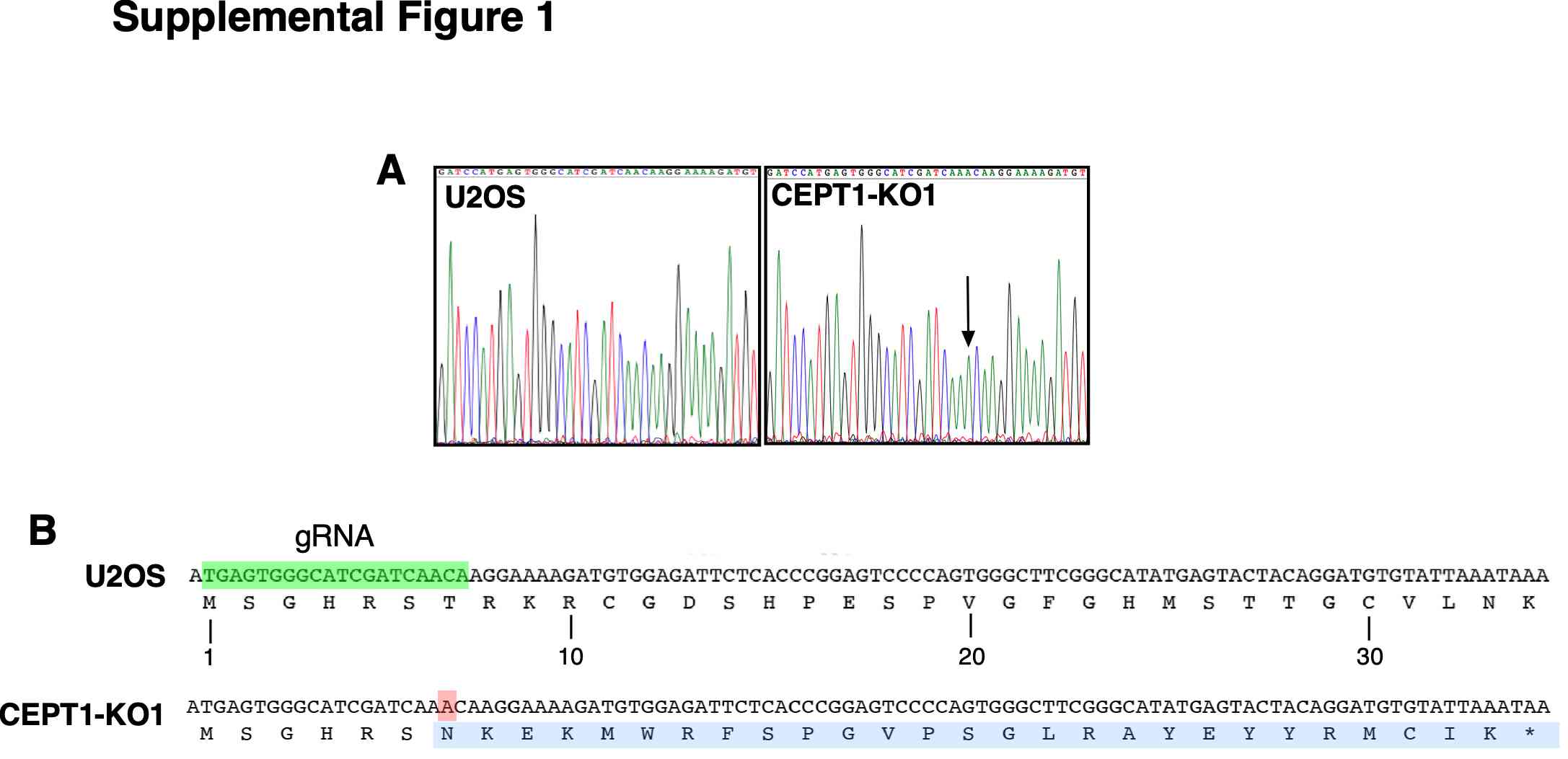
**

**Supplemental Figure 1**. CRISPR knockout of *CEPT1*. (A) Sanger sequencing tracing of genomic PCR products from U2OS and *CEPT1*-KO1 cells showing a single A insertion (arrow) at the gRNA cut site. (B) A comparison of exon 1 sequences from U2OS and *CEPT1*-KO1 cells showing the gRNA site (green) and the nucleotide insertion (red) that introduced a frame shift and stop codon (blue) in *CEPT1*-KO cells. CEPT1-KO2 cells had the same insertion and frame shift.

**
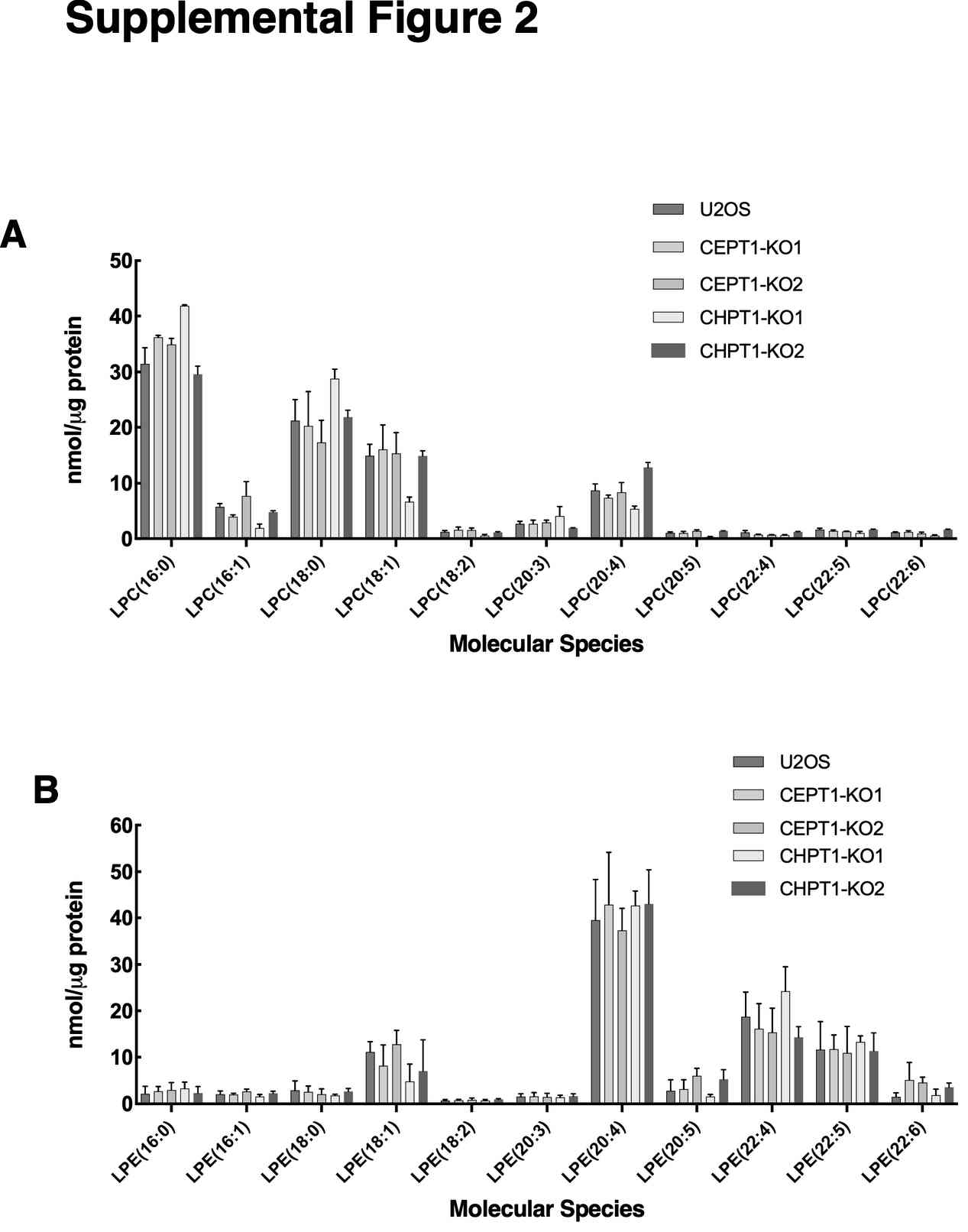
**

**Supplemental Figure 2**. Molecular species composition of lyso-PC and -PE in CHEPT1- and CHPT1-KO cells. The major molecular species of Lyso-PC (LPC, panel A) and lyso-PE (LPE, panelB) were quantified and expressed relative to cell protein. Results are the mean and SD of 3-6 biological replicates

**
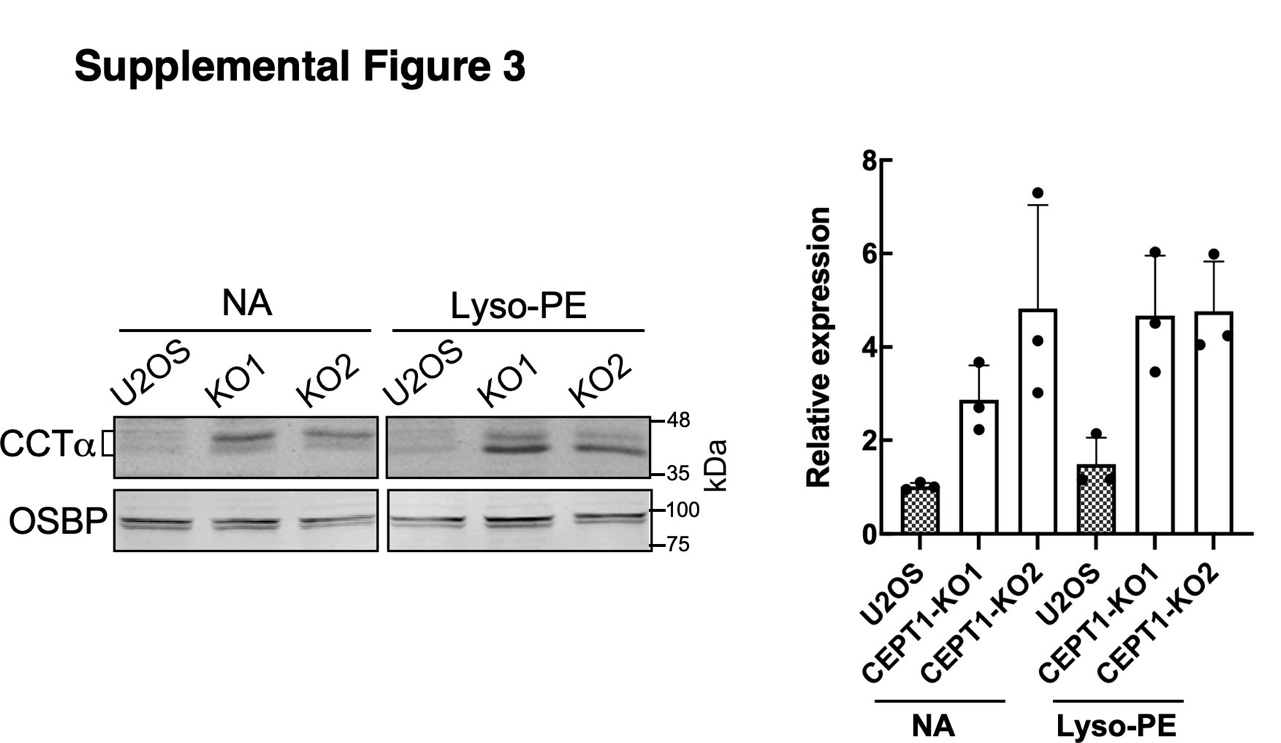
**

**Supplemental Figure 3**. Lyso-PE does not affect CCTα expression in *CEPT1*-KO cells. Cells were treated with 50 mM lyso-PE fore 24 h and CCTα expression was quantified from immunoblots as described in the legend to Fig. 6. Results are the mean and SD of 3 biological replicates

**
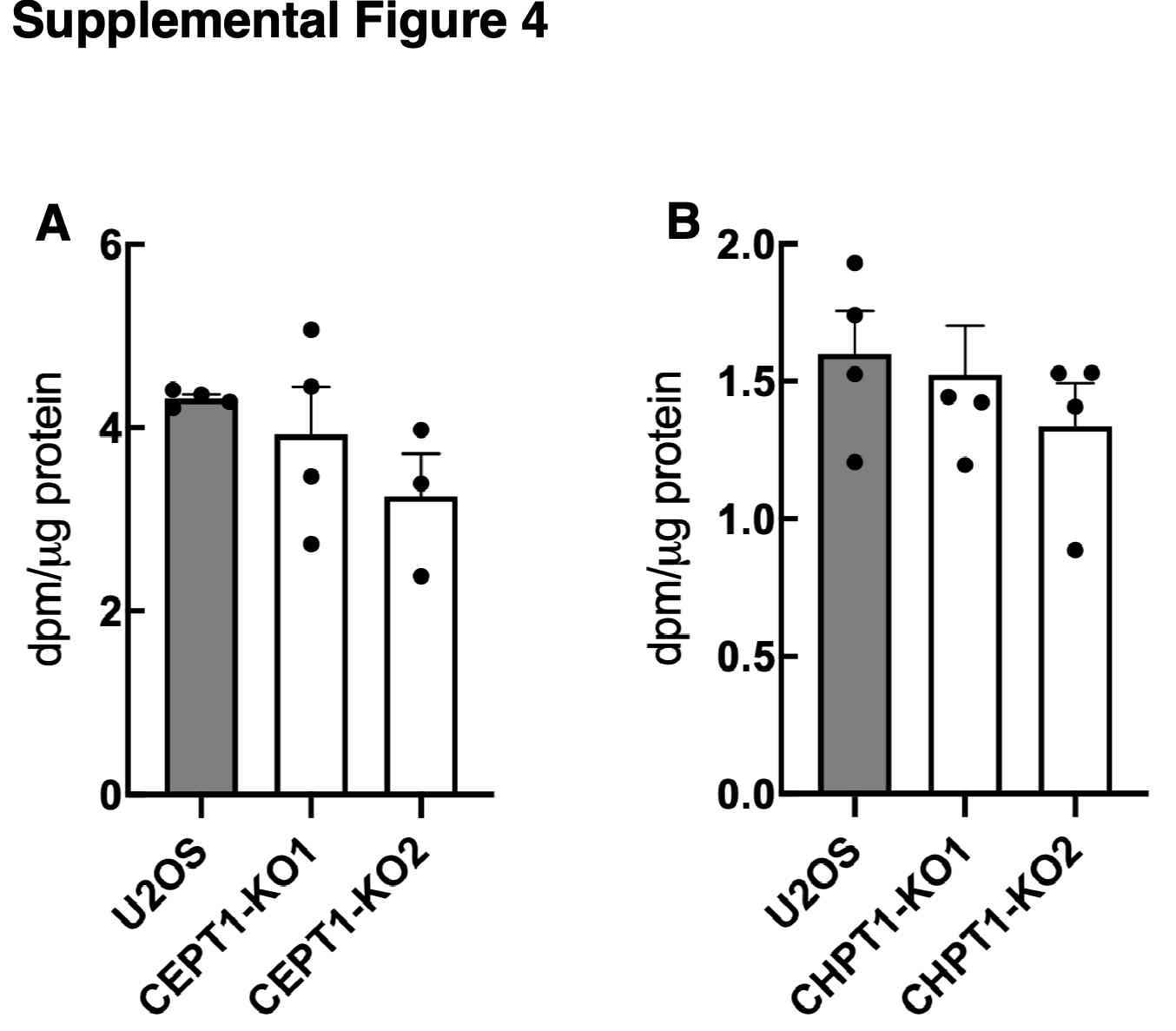
**

**Supplemental Figure 4**. Cholesterol ester synthesis is unaffected in *CEPT1*-KO and *CHPT1*-KO cells. Cells were labelled with 100 μM [^3^H]oleate (65 nCi/ml) for 4 h and incorporation into CE was measured and normalized to cell protein (results are the mean and SD of 3 biological replicates).

**Supplemental Table 1**. Sequence of PCR primers

| **Gene** | **Primers** | |
| --- | --- | --- |
| ***PGK1*** | forward | GACCTAATGTCCAAAGCTGAGAA |
|  | reverse | CAGCAGGTATGCCAGAAGCC |
| ***PCYT1A*** | forward | GCCCTATGTCAGGGTAACTATGG-3’ |
|  | reverse | GCGTGACCAGAGTGAAATAAGT |
| ***PCYT1B*** | forward | ATGCAAGAGCCCTTATGCAAG |
|  | reverse | GCTTCGTATCTCTCGGCTTCATT |
| ***CEPT1*** | forward | ATGTGGAGATTCTCACCCGGA |
|  | reverse | TCTTCTAGCCGCTTTAGTTGGT |
| ***CHPT1*** | forward | TTGCCGCTCGCTTAGGAAC |
|  | reverse | GCCTGAAACATAAGTCTGCCAAT |
| ***GAPDH*** | forward | CTGGGCTACACTGAGCACC |
|  | reverse | AAGTGGTCGTTGAGGGCAATG |
